# Spatial Chemical Stimulation Control in Microenvironment by Microfluidic Probe Integrated Device for Cell-Based Assay

**DOI:** 10.1371/journal.pone.0168158

**Published:** 2016-12-08

**Authors:** Masayuki Horayama, Kenta Shinha, Kazuya Kabayama, Teruo Fujii, Hiroshi Kimura

**Affiliations:** 1 Department of Mechanical Engineering, Tokai University, Hiratsuka, Kanagawa, Japan; 2 Department of Chemistry, Osaka University, Toyonaka, Osaka, Japan; 3 Micro/Nano Technology Center, Tokai University, Hiratsuka, Kanagawa, Japan; 4 Institute of Industrial Science, The University of Tokyo, Meguro, Tokyo, Japan; Universita del Salento, ITALY

## Abstract

Cell—cell interactions play an important role in the development and function of multicellular organisms. To investigate these interactions in detail, it is necessary to evaluate the behavior of a cell population when the minimum number of cells in the population is stimulated by some chemical factors. We propose a microfluidic device integrated with microfluidic probe (MFP) functionality; this device is capable of imparting a chemical stimulus to cells within a microenvironment, for cell-based assays. The device contains MFP channels at the walls of the cell culture microchannels, and it can control a localized chemical stimulation area at the scale of a single cell to a few cells using MFP fluid control in a microspace. The results of a finite element method-based simulation indicated that it is possible to control the chemical stimulation area at the scale of a single cell to a few cells by optimizing the MFP channel apex width and the flow ratio. In addition, localized cell staining was demonstrated successfully using a spatial chemical stimulus. We confirmed the device functionality as a novel cell-based assay tool. We succeeded in performing localized cell collection using this method, which suggested that the single cell analysis of a cell monolayer that is subjected to a specific chemical stimulus is possible. The method proposed in this paper can contribute significantly to the fields of cell biology and drug development.

## Introduction

Various cell dynamics such as proliferation, differentiation, and movement occurring *in vivo* at the scale of a single cell to a cell population are affected by the microenvironment surrounding the cells, including the interactions between cells. When performing cell-based assays *in vitro* to elucidate these cell dynamics, it is necessary to meticulously control the microenvironment surrounding the cells. However, this microenvironmental control is difficult in conventional cell culture methods that use culture dishes and well plates. To address these challenges, studies have commonly employed microfluidics technology within the research fields of μTAS (Micro Total Analysis Systems) and MEMS (Micro Electro Mechanical Systems)[1–4]. Gradient generators can be used to control the environment surrounding the cells by forming a concentration gradient of a humoral factor within microchannels, using laminar flow[5–8]. Such control methods, which use microfluidics technology, are widely employed in the field of bioscience, resulting in high throughput of cell-based assays[9]. These methods use laminar flow and have high spatial resolution in the vertical flow direction, with respect to spatial control. However, the methods have low spatial resolution in the horizontal flow direction, making cell assays at a single cell scale difficult.

A microfluidic probe (MFP) has been proposed for improving the spatial resolution of humoral factor stimulation control within cell culture environments[10–14]. MFPs have two microchannels, which are located adjacently across a gap of the order of tens of microns. A solution containing a humoral factor is injected into one channel, and before the humoral factor can disperse, it is suctioned from the other channel at a flow rate that is higher than the injection flow rate. As a result, localized chemical stimulation areas are formed at a single cell scale and a cell population scale.

Furthermore, a microfluidic device that can apply mechanical stimulation such as shear stress[15] or mechanical tension[16–18] to cells has been proposed for cell cultures. It has been reported that replicating the *in vivo* environment using this stimulation causes the cell dynamics to approach an *in vivo* state. It is conceivable that by additional spatial control of chemical stimulation using an MFP function in the cells that exhibit *in vivo*-like dynamics because of the mechanical stimulation that is applied using the microfluidic device, the cell dynamics that could not be explained using conventional cell-based assay techniques could be elucidated. However, the MFPs proposed previously are simple devices for open-culture systems like conventional cell culture dishes, and MFP functionality has not been integrated into microfluidic devices with closed-culture channels.

In this study, we developed a microfluidic device integrated with MFP functionality; this device capable of imparting a chemical stimulus to the cells within a microenvironment. The device contains MFP channels at the walls of cell culture microchannels, and it can control a localized chemical stimulation area at the scale of a single cell and a few cells using MFP fluid control in a microspace. Integrating cell culture and assay functions in a microfluidic device results in increased precision of control, economy of space, and elimination of contamination risk. Additionally, the device allows bright field observation of cells during chemical stimulation because there is no equipment that blocks transmitted light at the top of the stimulation area.

This paper reports the configuration of the proposed MFP integrated device and the results of a finite element method (FEM)-based simulation and a fluid control experiment that were performed to investigate the functionality of the device. The results of the simulation indicated that it is possible to control the chemical stimulation area at the scale of a single cell to a few cells by optimizing the MFP channel apex width and the flow ratio. In addition, localized cell staining and single cell collection were demonstrated using spatial chemical stimulus control, and we confirmed the device its functionality of as a novel cell-based assay tool.

## Materials & Methods

### Design and fabrication of the MFP integrated device

**Fig 1:**
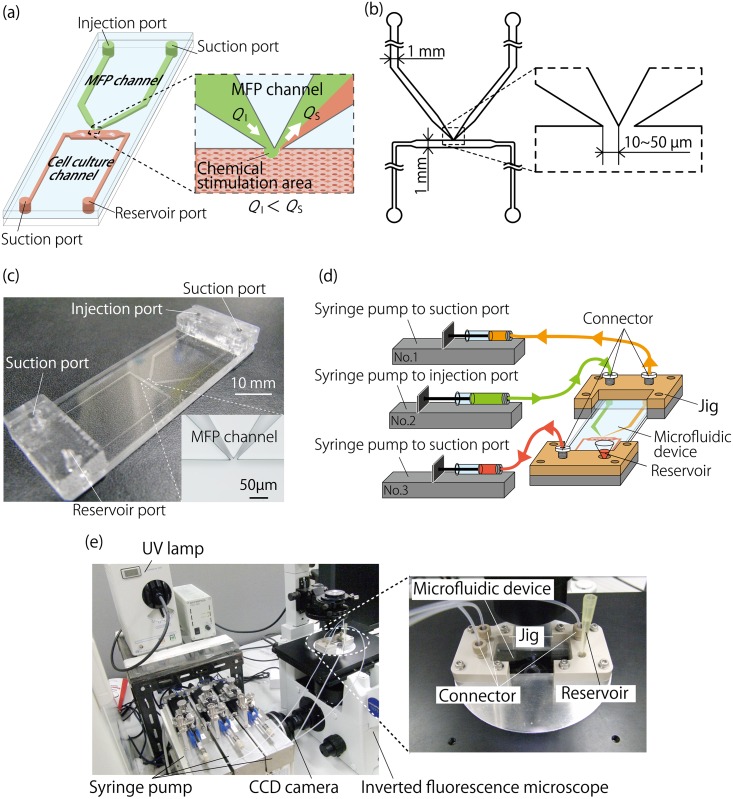
Conceptual diagram of the MFP integrated device. (a): MFP integrated device design. The device consists of an MFP channel (indicated in green) and a cell culture channel (indicated in red). To form a localized chemical stimulation area using the device, flow rates are set such that *Q*_*I*_ *< Q*_*S*_. (b): Scale diagram of the channel section of the MFP integrated device. The main portion of each MFP channel and the cell culture chamber have a channel width and height of 1 mm and 100 μm, respectively. Optimal values of the MFP channel apex width were investigated using an FEM-based simulation for values of 10–50 μm. (c): Device made from PDMS using photolithography. Two ports are fabricated at each microchannel in this device. In addition, a thick PDMS block is bonded to the locations of the ports for installation in a jig. (d): The device with jigs connected to syringe pumps. Syringe pump No. 1 and No. 2 are connected to the MFP channels, and are used for fluid control in the chemical stimulation area. Syringe pump No. 3 is connected to the suction port of the cell culture channel, and is used for cell culturing, solution replacement, and other related activities. (e): Experimental setup. The device was installed onto the stage of an inverted fluorescence microscope with a CCD camera.

The microfluidic device was installed with two MFP channels for localized chemical stimulation and a cell culture channel for culturing cells (Fig 1(a)). One of the MFP channels was for injecting the solution containing a humoral factor and the other was for suctioning the solution. The cell culture channel consisted of a cell culture chamber and a microchannel for cell inoculation and media replacement. The MFP and cell culture channels were connected at the apex of the MFP channels, which were joined without any gap between them and the wall surface of the cell culture chamber. The apex width of the MFP channels was designed to be 10–50 μm to achieve the formation of a high-resolution chemical stimulation area. The MFP channels and the cell culture chamber had a width and height of 1 mm and 100 μm, respectively, for each case (Fig 1(b)).

The microfluidic device was fabricated using conventional photolithography and soft lithography[19]. A Cr mask substrate for photolithography, which was used to fabricate the microchannels, was designed using CAD software (AutoCAD, Autodesk, USA), and fabricated using a micro-pattern generator (μPG101, Heidelberg instruments, Germany). A negative photoresist (SU-8 3050, MicroChem) was spin coated onto the Cr mask substrate, and after baking, the channel pattern was formed on the substrate using ultraviolet lamp exposure. The substrate was used as the master mold for polydimethysiloxane (PDMS, SILPOT 184, Dow Corning Toray), which was the material of the microfluidic device. A 10:1 mixture of PDMS and a polymerization agent was poured onto the master mold, and heat cured in an oven at 75°C for 2 h. The cured PDMS was peeled from the master mold to produce a PDMS chip to which the channel pattern was transferred. Microchannel ports, which had a diameter of 2 mm, were fabricated on the PDMS chip using a trepan (BP-L20K, Kai). The surfaces of the PDMS chip and a glass plate were activated using a plasma cleaner (PDC-32G, Harrick Plasma) and then bonded together to fabricate the microfluidic device. Furthermore, PDMS blocks were bonded at the locations of the ports using plasma bonding to set the device into a jig, as described below. The fabricated device is shown in Fig 1(c).

### Microfluidic control setup and method

The microfluidic device was set in a jig that was made from a machined PEEK. To control the MFP functionality, the two MFP channel ports and one of the cell culture channel ports were connected to three independent high-resolution syringe pumps (MFS-SP3, Microfluidic System Works) using Fluorinated Ethylene Propylene (FEP) tubes (OD: 1/16 inch and ID: 1/100 inch, Upchurch Scientific). A reservoir for stocking a medium was installed at the other port of the cell culture channel.

The flow rate in the MFP channels was controlled using syringe pump No.1 and No. 2, as shown in Fig 1(d), which were connected to the MFP channel ports. Using these pumps, the solution containing a humoral factor was injected at a flow rate *Q*_*I*_ into the injection port of the MFP channel, and was suctioned at a flow rate *Q*_*S*_ from the suction port of the MFP channel, as shown in Fig 1(a). It was possible to suppress the diffusion of the humoral factor in the solution by taking *Q*_*I*_ < *Q*_*S*_, thereby forming a high-resolution localized exposure area for the humoral factor within the cell culture chamber. At this time, the amount of the medium corresponding to the difference between *Q*_*S*_ and *Q*_*I*_ was discharged from the cell culture channel, allowing for a sufficient amount of the medium to be stocked in the reservoir. In addition, syringe pump No. 3 was used for suctioning the solution from the reservoir, for cell inoculation and medium replacement in the cell culture channel. The device, together with the jig, was installed onto a transparent hotplate (37°C) on the stage of an inverted fluorescence microscope (IX71, Olympus) with a charge-coupled device (CCD) camera (DP72, Olympus), as shown in Fig 1(e).

### Analysis of chemical stimulation area using FEM-based simulation

To achieve a resolution at the scale of a single cell to a few cells for the chemical stimulation area, it is necessary to understand the relationship between this area and the ratio of *Q*_*I*_ and *Q*_*S*_, referred to as the flow ratio, and to investigate the apex width of the MFP channels. In this experiment, we optimized the apex width and the flow ratio of the MFP channels using the general-purpose physics simulation software COMSOL Multiphysics (COMSOL), which is based on the FEM.

In this simulation, five values were taken for the flow ratios (*Q*_*I*_:*Q*_*S*_ of 1:10, 2:10, 3:10, 4:10, and 5:10). The apex width of the MFP channels was set to be 10, 20, 30, 40, and 50 μm. Fluorescein sodium salt was used as the humoral factor, which had a diffusion coefficient of *D* = 0.6 × 10^−5^ cm^2^/s and a solution concentration of 5.0 μM. The chemical stimulation area was measured from the images, obtained from the simulation results, using image-processing software (ImageJ, NIH) in steady-state under each condition with a threshold concentration, which was of 20% of the original solution concentration. Fluid shear stress was calculated using the equation *τ* = 6*μQ*/*bh*^2^, where *μ* is the medium viscosity (Pa·s), *Q* is the estimated average flow rate (m^3^/s), *b* is the channel width (m), and *h* is the channel height (m)[20].

### Evaluation of MFP functionality of the device

An experiment was performed using the fabricated microfluidic device to verify the validity of the simulation results. The device was installed on the stage of the microscope, and the ports were connected to the three syringe pumps and the reservoir, as described previously. *Q*_*I*_ was set to be 0.1, 0.2, and 0.5 μL/min, while *Q*_*S*_ was set to be 1.0 μL/min. In the experiment, 5 μM uranine (fluorescein sodium salt, F0096, Tokyo Chemical Industry) was used as the humoral factor model, and fluorescence images were captured within the channel using the microscope. The chemical stimulation area in the channel was measured quantitatively from the fluorescence images using ImageJ, with a threshold value of 20% of the concentration of the original solution.

### Cell inoculation and culture

In the experiment, CHO (Chinese Hamster Ovary) cells cultured using conventional methods were used to evaluate the device. These cells were obtained from the JCRB Cell Bank and cultured at 37°C in an incubator, in a humidified atmosphere containing 5% CO_2_. The culture medium was Ham’s F-12 (087–08335, Wako Pure Chemical Industries) supplemented with 10% fetal bovine serum (FBS, Bio West), 1% non-essential amino acid solution (11140–050, Thermo Fisher), and 1% antibiotic antimycotic solution (161–23181, Wako).

Cell inoculation and culture were performed within the device according to the following procedure. First, the cell culture channel was coated with an extracellular matrix by introducing 30 μg/mL of laminin solution (120–05751, Wako Pure Chemical Industries) to the reservoir via suction control using the syringe pump (No. 3 in Fig 1(d)) at the suction port. The channel was coated with the laminin solution by placing the device in the incubator for 30–60 min. After the coating process, the laminin solution inside the channel was removed and the channel was washed using phosphate buffered saline (PBS) through the reservoir. The cells were seeded at a density of 2.3 × 10^5^ cells/cm^2^ by introducing a cell suspension to the reservoir via suctioning using syringe pump No. 3. Then, the device was placed in the incubator for 2–3 h to allow the cells to adhere to the bottom surface of the cell culture channel. After cell adhesion, the culture medium stored in the reservoir was suctioned at a flow rate of 0.1 μL/min using syringe pump No. 3 for a perfusion culture.

### Spatial chemical stimulation of cells

In the first experiment, the localized chemical stimulation of the cells in the cell culture chamber was investigated to study the functionality of the device. 100 μM Rhodamine B (R0040, Tokyo Chemical Industry) was injected using the syringe pump (No. 2) connected to the injection port of the MFP channels, and it was simultaneously suctioned using syringe pump No. 1. The cells in the channel were observed using the inverted fluorescence microscope while changing the flow ratios (*Q*_*I*_:*Q*_*S*_) every 10 s to be 0.1 μL/min:1.0 μL/min, 0.2 μL/min:1.0 μL/min, and 0.5 μL/min:1.0 μL/min.

In the second experiment, an investigation was performed to identify if the cells in the cell culture chamber could be collected site-selectively using localized stimulation with a digestive enzyme solution. The device was seeded with cells according to the procedure described previously. A 0.5% w/v trypsin/1mM EDTA.4Na solution with phenol red (206–17291, Wako) was injected at a flow rate (*Q*_*I*_) of 0.2 μL/min from the injection port of the MFP channels, and it was simultaneously suctioned at a flow rate (*Q*_*S*_) of 1.0 μL/min. 5.0 μM uranine was added to the sample solution to make the chemical stimulation area visible. In this manner, the cells were exposed to a site-localized trypsin solution and collected after desquamation.

## Results and Discussion

### Analysis of chemical stimulation area using FEM-based simulation

**Fig 2:**
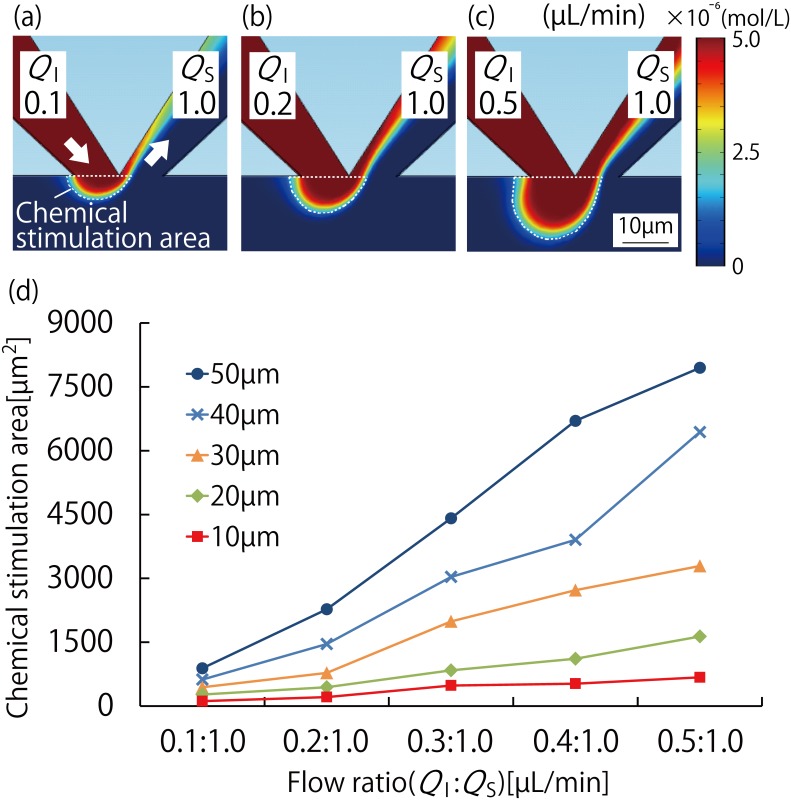
Fluid analysis simulation results using COMSOL Multiphysics for evaluation of the MFP functionality of the MFP integrated device. (a)–(c): Calculation results for the chemical stimulation area when the MFP channel apex width was 10 μm and the flow ratios (*Q*_*I*_:*Q*_*S*_) were (a) 0.1 μL/min:1.0 μL/min, (b) 0.2 μL/min:1.0 μL/min, and (c) 0.5 μL/min:1.0 μL/min. The chemical stimulation area increased with the flow ratio. (d): Graph showing the relationship between MFP channel apex width and flow ratio. The MFP channel apex width was taken as 10, 20, 30, 40, and 50 μm. When the apex width increases, the increase in the chemical stimulation area with the flow ratio is larger.

The simulation was performed to evaluate the fluid behavior for different flow ratios in the MFP channels, and to optimize the apex width of these channels. In the experiment, the area of the cell culture chamber that had a humoral factor concentration of at least 20% was defined as the chemical stimulation area. The simulation results when the apex width of the MFP channels was 10 μm and the flow ratios (*Q*_*I*_:*Q*_*S*_) were 0.1 μL/min:1.0 μL/min, 0.2 μL/min:1.0 μL/min, and 0.5 μL/min:1.0 μL/min are shown in Fig 2(a), 2(b), and 2(c), respectively. These results showed that the semicircular chemical stimulation area broadens with an increase in the flow ratio. The formation of a concentration gradient owing to substance diffusion was observed in an area that was at the boundary surface of the cell culture chamber side of the chemical stimulation area. We found that there was no significant change in the distance of the chemical stimulation area from the boundary. However, it broadened with an increase in the flow ratio, for the values of the flow ratio that we considered.

In this simulation, we also calculated the magnitude of and the shear stress on the stimulation area for 1/10 and 10 times value of the original flow ratio (0.5 μL/min:1.0 μL/min) when the apex width was 10 μm. For the original flow ratio, the stimulation area where the concentration was more than 90% was 47% of the total stimulation area, and the shear stress was 0.3 Pa. When the value was 1/10 of the original, the stimulation area where the concentration was more than 90% was only 16% of the total stimulation area. This means that a larger boundary surface appeared owing to a longer diffusion time because of lower flow velocity as compared to the case with the original value. When the value was 10 times of the original, the stimulation area where the concentration was more than 90% was 72% of the total stimulation area, and the average shear stress was more than 3.8 Pa. Keane et al. reported that there are no morphological changes in CHO cells when the shear stress is between 0.005 to 0.8 Pa. However, extremely high shear stress affects cellular functions such as glucose uptake and lactate production rates. Therefore, the original values of the flow ratios (of 0.1 μL/min:1.0 μL/min, 0.2 μL/min:1.0 μL/min, 0.3 μL/min:1.0 μL/min, 0.4μL/min:1.0μL/min and 0.5 μL/min:1.0 μL/min) were used in the experiments for demonstrating the device functions.

We considered that the apex width of the MFP channels affects the dimensions of the stimulation area. The apex width of 10 to 50 μm used in this study was decided based on the values in literature, e.g., 20 μm[13] and 50 μm[14]. As shown in Fig 2(d), the chemical stimulation area increased almost linearly with the flow ratio regardless of the apex width of the MFP channels. In addition, the slope of the fitted curve increased with the apex width of the MFP channels. Based on these results, it can be concluded that the chemical stimulation area has a high correlation to the flow ratio and the apex width of the MFP channels, and that control is possible over a broad range of scales by varying these parameters. Typically, the scale of a single cell is less than 400 μm^2^, although this depends on the cell type. The simulation results indicated that control of the chemical stimulation area at high spatial resolution, applicable to the scale of a single cell and a few cells, is possible at an MFP channel apex width of 10 μm, thereby satisfying the objective of this study.

### Evaluation of MFP functionality of the device

**Fig 3:**
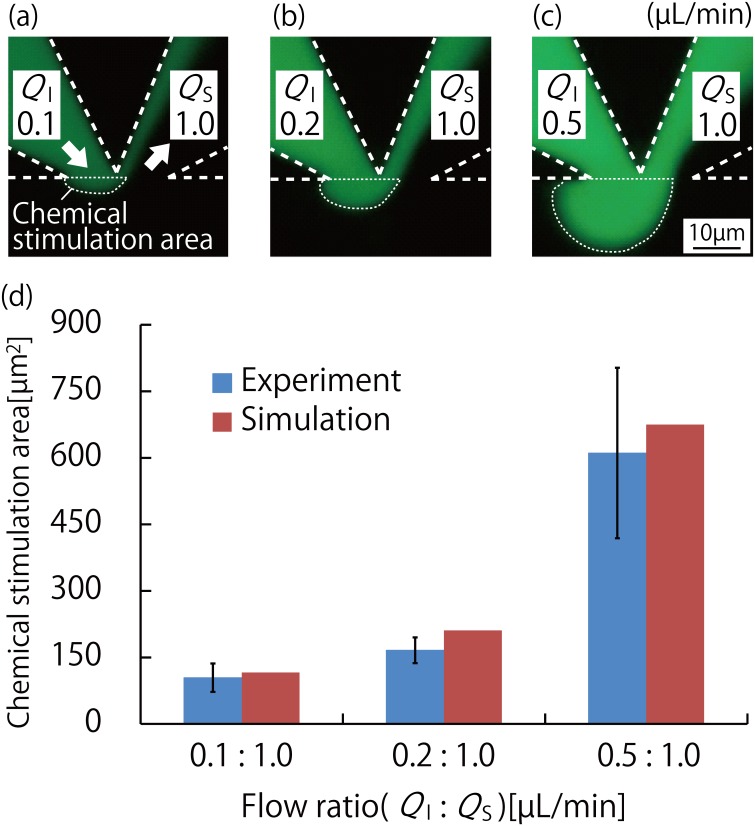
Results of the fluid control experiment for the evaluation of MFP functionality of the MFP integrated device. (a)–(c): Fluorescence images of the chemical stimulation area and its vicinity when the MFP channel apex width is 10 μm, and fluid control is for MFP channel flow ratios (*Q*_*I*_:*Q*_*S*_) of (a) 0.1μL/min:1.0 μL/min, (b) 0.2 μL/min:1.0 μL/min, and (c) 0.5 μL/min:1.0 μL/min. Uranine, a green fluorescent dye, was used as the humoral factor model. The chemical stimulation area increased with the flow ratio. (d): Graph comparing the results of the COMSOL Multiphysics simulation and the flow control experiment performed using the fabricated device. Both results were almost identical and the difference between them was approximately 10%. Means ± S.D., n = 3.

Based on the simulation results described in the previous section, a device having an MFP channel apex width of 10 μm was fabricated, and used in the fluid control evaluation experiment. The fluorescence images of the stimulation area for three flow ratios (*Q*_*I*_:*Q*_*S*_ of 0.1 μL/min:1.0 μL/min, 0.2 μL/min:1.0 μL/min, and 0.5 μL/min:1.0 μL/min) are shown in Fig 3(a), 3(b), and 3(c), respectively. The semicircular chemical stimulation area broadened with increase in the flow ratio. Fig 3(d) shows the data from the quantitative measurements of these images, which were carried out using ImageJ, and the data from the simulations carried out under the same conditions as described in the section titled “Analysis of chemical stimulation area using FEM-based simulation.” The difference between the results from the experiment and the simulation was less than 10%, which showed that the results obtained using both methods were nearly equal. This validates the results of simulation and proves that the channel shape and fluid operations of the device can be optimized using simulations. Based on these results, it can be concluded that the chemical stimulation area at the scale of a single cell to a few cells can be controlled using the high-resolution fluid control technique of the proposed device.

### Spatial chemical stimulation of cells

Using the fluid control method established by the results described above, we performed an experiment to assess if spatial chemical stimulation of cells was possible using the device. To perform this experiment, CHO cells were cultured in the cell culture chamber ahead of time, forming a monolayer. Rhodamine B, which can stain cell membranes, was used as the humoral factor model. We evaluated whether a solution of Rhodamine B could impart a spatial chemical stimulus to the cell monolayer using the MFP functionality.

**Fig 4:**
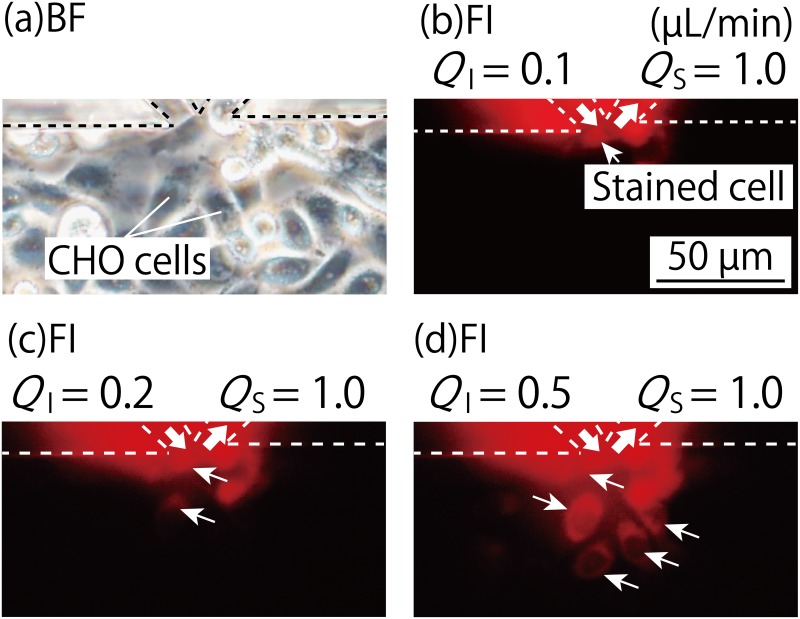
Images within the cell culture chamber when CHO cells were chemically stimulated using the MFP functionality of the MFP integrated device. (a): Bright-field image of CHO cells in the vicinity of the MFP channel apex in the cell culture chamber. (b)–(d): Fluorescence images of spatial cell membrane staining of the CHO cells in the cell culture chamber using a Rhodamine B solution. The flow ratios (*Q*_*I*_:*Q*_*S*_) were (b) 0.1 μL/min:1.0 μL/min at *t* = 0 s, (c) 0.2 μL/min:1.0 μL/min at *t* = 10 s, and (d) 0.5 μL/min:1.0 μL/min at *t* = 20 s. One to several cells was stained by exposure to the Rhodamine B solution as the chemical stimulation area changed with the flow ratio over time.

Fig 4 shows the cell states for three flow ratios (*Q*_*I*_:*Q*_*S*_ of 0.1 μL/min:1.0 μL/min, 0.2 μL/min:1.0 μL/min, and 0.5 μL/min:1.0 μL/min). When the flow ratio was 0.1 μL/min:1.0 μL/min, only one cell in the cell culture chamber, in the vicinity of the MFP channels, was stained with red fluorescence. When the flow ratio was 0.2 μL/min:1.0 μL/min, two or three cells were stained owing to the broadening of the stimulation area, and for the flow ratio of 0.5 μL/min:1.0 μL/min, five cells were stained owing to further expansion of the stimulation area. The fluorescent staining process was performed sequentially, which indicated the possibility of temporal changes in the control over the chemical stimulation area. Based on these results, it can be concluded that the fabricated device is capable of controlling the spatial chemical stimulation area at the scale of a single cell to a few cells at high spatial resolution.

**Fig 5:**
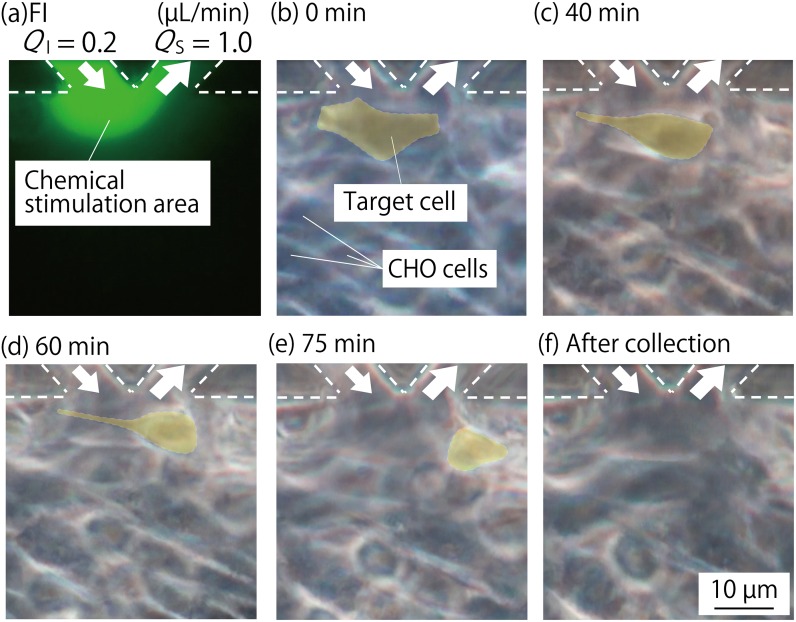
Images within the cell culture chamber when a CHO cell was exposed to trypsin by controlling the chemical stimulation area using the MFP functionality of the MFP integrated device. The flow ratio (*Q*_*I*_:*Q*_*S*_) was set to be 0.2 μL/min:1.0 μL/min during the experiment. (a): Fluorescence image showing the chemical stimulation area in the microchannel, using fluorescein sodium salt. (b)–(f): Bright-field images of cells when exposing a single cell in the cell culture chamber to the trypsin solution, using the MFP functionality to collect the cell. The cell present in the chemical stimulation area, shown in (b), lost its adhesion ability over time, and contracted to reach the states shown in (c)–(e) after 40–75 min. It completely detached from the substrate, shown in (f), and was collected from the device through the suction port inside the MFP channel. At this time, the cells surrounding the collected cell were not affected by trypsin.

Fig 5 shows the case when the cells within the device were collected locally by controlling the chemical stimulation area. A cell (Fig 5(b)) present in the chemical stimulation area, identified using a fluorescence image, (Fig 5(a)) lost its adhesion ability over time and contracted. After 40–75 min, a portion of the cell had detached from the substrate (Fig 5(c), 5(d), and 5(e)), after which it detached completely (Fig 5(f)). At this time, the cells surrounding the detached cell were not affected by the trypsin. The detached cell could be collected through the suction port.

Cells were present at the bottom of the MFP channels. The adhesion and migration of the cells in the channels could not be controlled because collagen was coated as the extracellular matrix on the surface of the microchannel, including the MFP region. However, the thickness of adhesive CHO cells is approximately 2 μm[21], whereas the thickness of the microchannel is 100 μm. The presence of the cells in the MFP channel could be neglected because their thickness is significantly less than that of the microchannel. Moreover, only laminar flow occurs in the microchannels owing to an extremely low Reynolds number (Re) in the channels. We consider that the presence of the cells does not affect the fluid dynamics under the experimental conditions.

The threshold value of the solution concentration, i.e. 20%, for the calculation and evaluation of the stimulation area has been decided according to the preliminary experiment carried out using the trypsin solution. We evaluated the threshold value of the effective concentration of the trypsin solution based on the conventionally used concentration (0.5% w/v trypsin solution). The result suggested that a 20% dilute trypsin solution (0.1% w/v trypsin solution) affected adhesion of CHO cells. Hence, the threshold of 20% was applied in this study. Although effective thresholds of chemical concentration might be due to chemical characteristics, the experiments carried out using chemicals such as uranine, Rhodamine B, and trypsin succeeded. Thus, we concluded that the threshold value was reasonable.

In the single cell collection experiment, the trypsin exposure time of over 40 min is longer than that of conventional plate assays. We consider that this is because of the temperature during the assay. Although the device was placed onto the hotplate, it might not be able to control the temperature in the cell culture chamber as much as in a 37°C incubator. In fact, the trypsin exposure time of over 40 min might be needed to peel the cells off during the pilot test, using plates on the hotplate to evaluate the threshold concentration of trypsin solution. We would be unable to do this additional study because of equipment limitations. However, we can still conclude that this is the first demonstration of single cell collection from a confluent cell monolayer, and not from the sparse condition that was used by Juncker et al. [13]. Additionally, the device allowed for bright field observation of cells during chemical stimulation because there is no equipment that blocked the transmitted light at the top of the stimulation area, as shown in Fig 5.

In recent years, it has been reported that individual cells in a cell population have differences in gene expression at the molecular level, protein expression patterns, etc., even if they are of the same type[22]. In other words, when biochemical analysis is performed at the cell population scale, only the data that has been averaged over individually varying parameters can be obtained. In response, techniques in molecular biology and engineering have been developed to perform single cell analysis, which are capable of analyzing the parameters that are different for each cell. To perform single cell analysis, it is necessary to collect a single cell, and then perform the operations that are required to obtain single cell parameters via biochemical processes. In the field of μTAS, a microfluidic device that integrates the biochemical analysis of the genes and proteins from a cell that is collected downstream of an MFP has been reported[23]. This technique can also be applied to the proposed device. Using this technique, there is high potential for achieving a cell-based assay that is capable of an even more detailed analysis. This could theoretically be accomplished by imparting a stimulus to a cell cultured in an environment that has been spatially controlled with precision within a microchannel, and then collecting that cell.

## Conclusion

In this paper, we propose an MFP-integrated device as a novel cell-based assay tool, and establish a fluid control method capable of delivering spatial chemical stimuli to a cell within a microchannel. Integrating an MFP function with a microfluidic device allows for controlling a stimulation area at high resolution, and obtaining bright-field images during the assay.

Meanwhile, the method proposed in this paper for integrating MFP functionality into a microchannel has low freedom of spatial control owing to its two-dimensional configuration, and, at present, having a chemical stimulation area that is limited to the vicinity of the channel wall. However, it is conceivable that freedom of spatial control can be increased by forming MFP channels in the ceiling of the microchannel to integrate MFP functionality in a three-dimensional manner. Furthermore, the freedom of spatial control can be increased significantly by applying MEMS integration techniques to configure an array of MFP functionalities. Although it is necessary to overcome such issues to improve practical usability, the method proposed in this paper can contribute significantly to the fields of cell biology and drug development.
